# Childhood nutritional stress and later-life health outcomes in medieval England: Evidence from incremental dentine analysis

**DOI:** 10.1126/sciadv.adw7076

**Published:** 2025-07-30

**Authors:** Sharon N. DeWitte, Julia Beaumont, Brittany S. Walter, Jacqueline R. Towers, Emily J. Brennan

**Affiliations:** ^1^ Institute of Behavioral Science, University of Colorado Boulder, CO 80309, USA.; ^2^ Department of Anthropology, University of Colorado, Boulder, CO 80309, USA.; ^**3**^ School of Archaeological and Forensic Sciences, University of Bradford, Bradford, UK.; ^4^ Defense POW/MIA Accounting Agency, Department of Defense, Offutt AFB, Bellevue, NE 68113, USA.; ^**5**^ Center for Integrative and Experiential Learning, University of South Carolina, Columbia, SC 29208, USA.

## Abstract

Numerous studies have revealed links between prenatal/early-life stress and elevated morbidity and risks of mortality later in life. Given the number of subsistence crises in medieval England, this study uses stable isotopic, demographic, and paleopathological data from human skeletal remains (*n* = 275) to assess associations between early-life nutritional stress and health outcomes before, during, and after the Black Death in London and rural Lincolnshire approximately 1000 to 1540 CE. Our results suggest that survivors of early-life nutritional stress were resilient with respect to causes of death in childhood, adolescence, and early adulthood. Ultimately, however, early-life nutritional stress was associated with the presence of skeletal biomarkers of stress and elevated mortality in middle and late adulthood in the medieval period, consistent with models from developmental biology. We find that the prevalence of nutritional stress increased before the Black Death and decreased afterward. Understanding the long-term consequences of early-life nutritional stress can offer insights on the health trajectories of historical populations.

## Teaser

Childhood malnutrition may have led to poor adult health in medieval England and became less common after the Black Death.

## INTRODUCTION

### Nutritional stress and subsistence crises in medieval England

The medieval period in England was replete with crises, including famines and plague epidemics. Perhaps the most marked of these was the mid-14th-century CE pandemic of plague that is now often called the Black Death and was the first wave of the Second Pandemic of Plague. Evidence from historical documents and previous bioarchaeological research suggests that health, at least in London, declined before the Black Death arrived in England in 1348 CE and that following the epidemic, health improved in the City at least temporarily (*1*–*3*). These observed trends might have, respectively, been the result of nutritional deprivations particularly due to repeated famines before the Black Death and improved standards of living (including improved diet across all social strata) after the epidemic ended.

There were famines in England every 14 years on average in the 11th to 13th centuries CE, with a slight decrease in the 14th to 16th centuries CE; overall, there were approximately 7 famines per century or 10 years of famine per century from 1201 to 1600 CE (*4*). According to Alfani and Ó’Gráda (*5*), there was a concentration of famines across Europe in the decades just before the Black Death and relatively few for about two centuries afterward. These famines are often attributed to the climate changes associated with the Medieval Climate Anomaly and its end, with their effects exacerbated by social and economic factors [e.g., see (*6*)]. Nutritional status strongly influences health at the individual level, e.g., directly resulting in death from starvation or indirectly by increasing susceptibility to infectious disease during periods of food shortages (*7*, *8*) or by altering development in ways that produce poor health outcomes later in life, as detailed below. It can also affect health across generations via epigenetic modifications (*9*). Thus, exposure (or lack thereof) to malnutrition may have had both immediate health consequences for the individuals affected and intergenerational effects that shaped health across the medieval period in England.

Clarifying the possible effects of nutritional stress on health during the medieval period is potentially important for improving our understanding of variation in medieval plague experiences and outcomes, particularly from the perspective of syndemic theory. The concept of the syndemic recognizes that diseases are not simply biological and outcomes are not just the result of the interaction of a pathogen with a person’s immune system but rather reflect the synergistic effects of broader individual-level and societal structural conditions (*10*). To understand disease susceptibility and outcomes, we must pay careful attention to biological, environmental, physical, and social contexts—i.e., coinfections with multiple pathogens, malnutrition, wealth inequality, social marginalization, and other factors that shape exposures and vulnerability to disease and death. Historical plague outbreaks, such as the Black Death, provide an excellent opportunity for bioarchaeologists to engage with the concept of syndemics in a fruitful way (*11*–*13*). This is because several lines of evidence (bioarchaeological, paleoclimatological, biogeochemical, historical, and paleogenomic) across different regions can be used to examine multiple co-occurring social, biological, and environmental conditions that contributed to variations in disease exposure, severity, and outcomes at the individual and population levels. Further, it is increasingly clear that we need more information regarding the variable contexts in which medieval plague emerged as our understanding of mortality patterns and levels during the pandemic is gaining more nuance. For example, Izdebski *et al.* (*14*) reported, on the basis of paleoecological data, that there was spatial heterogeneity in the levels of mortality produced by the Black Death, which counters the assumption that the pandemic was equally devastating everywhere across Afro-Eurasia.

One thing that varied across pre–Black Death populations was the degree of population pressure and the existence or extent of subsistence crises. This variation and the links that have been made previously between famine and plague outbreaks (*15*, *16*) highlight the need to carefully examine the relationships between malnutrition and health in the context of historical plague.

In this study, we use stable isotope data from incremental samples of tooth dentine from 275 medieval individuals to assess the effect of severe nutritional stress in utero and during childhood on health outcomes later in life in medieval London and Lincolnshire, England. Because teeth form in a well-understood and robust manner during childhood and adolescence and do not remodel, we can use the patterns of change within the isotope ratios of carbon (δ^13^C) and nitrogen (δ^15^N) to investigate changes in diet and physiology over defined periods of life. More details about stable isotopic approaches to dietary and nutritional stress reconstructions in past populations are provided below. The approach we take in this study allows us to examine the effects of a specific stressor (nutritional stress) during development on later health outcomes. This is a major strength of our study and is in contrast with much of the existing scholarship in bioarchaeology that relies largely on skeletal biomarkers of unknown etiologies (and, for some biomarkers, unknown ages of exposures). By clearly identifying individuals who experienced nutritional stress during sensitive windows of development, our findings may be more clearly aligned with research on recent and living human populations that have experienced identifiable stressors (such as the Dutch Famine, as detailed below). Our general goals are to expand our understanding of the context of the emergence of the Black Death in England and health patterns in its aftermath and, more broadly, to contribute to the bioarchaeological examination of the developmental origins of health in past populations [e.g., (*17*–*24*)].

### Developmental stress

A growing body of evidence suggests that the intrauterine and early childhood environments can affect health throughout life, including health in adulthood decades later. Observations of the relationship between exposures to environmental stressors in utero/early in life and later-life health outcomes have led to the creation of the field of Developmental Origins of Health and Disease (DOHaD). The relationship between early-life stress and health later in life exists because the development of our organ and physiological systems [e.g., the hypothalamic-pituitary-adrenal (HPA) axis and the immune system] can be influenced by the nature, severity, and timing of exposures to stressors (*25*–*27*). The overarching mechanism producing these relationships is developmental phenotypic plasticity (*26*, *28*). Developmental phenotypic plasticity describes the production of different phenotypes from a single genotype as a result of adaptations during development in response to concurrent environmental conditions (*29*). Whether these adaptations are beneficial over the short-term or the long-term depends on a variety of factors, and thus plasticity can have variable implications for health and survival across an individual’s life span.

Because energetic resources are finite and must be allocated to competing metabolic demands, like tissue maintenance versus growth versus reproduction, the experience of stress during development can lead to trade-offs, such as the temporary cessation of growth and alterations of development in favor of short-term survival in the face of stressors like malnutrition (*30*). This can lead to impaired function or dysregulation of biological systems that ultimately harms health later in life. For example, as described by the thrifty phenotype hypothesis [which is aligned with the predictive adaptive response (PAR) model described below (*31*)], intrauterine undernutrition can stimulate growth restriction of the fetus via the development of insulin resistance, which favors short-term survival but may lead to diabetes and other conditions later in life, particularly if the postnatal conditions do not match prenatal conditions (e.g., an abundance of calories later in life following a developmental period characterized by scarcity) (*30*, *32*, *33*). Similarly, malnutrition can compromise the development of the immune system (*34*), producing increased susceptibility to infectious disease later in life.

Epigenetic modifications have also been proposed as mechanisms linking exposures to stressors during development and health outcomes later in life (*32*, *35*). Studies in humans and nonhuman animals have produced evidence of epigenetic modifications associated with developmental stress, and associations have been found between epigenetic variation and negative health outcomes in adulthood. For example, studies of living people have found epigenetic modifications among individuals exposed to prenatal or early-life nutritional stress that may be associated with increased risk of metabolic disease and other chronic conditions in adulthood [e.g., (*36*–*38*)].

### Models of the developmental origins of health

**Fig. 1.**
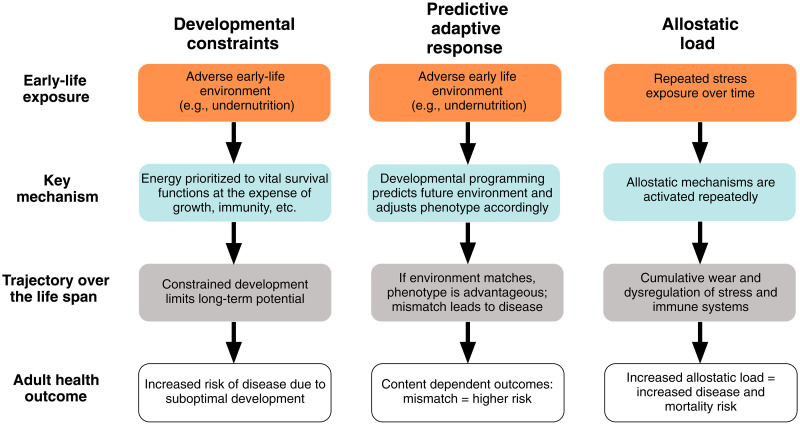
Conceptual comparison of three models within the developmental origins of health framework. Developmental constraints (early adversity limits optimal development), PAR (outcomes depend on match/mismatch between early and later environments), and allostatic load (cumulative stress leads to physiological dysregulation over time).

There are several models or frameworks regarding the developmental origins of health, and among these, the PAR, developmental constraints, and allostatic load models have been applied in numerous studies in human biology or bioarchaeology [e.g., (*19*, *20*, *22*, *31*, *39*, *40*)]. These three models are represented in Fig. 1.

According to the developmental constraints model, adverse environmental conditions stimulate a developing individual to allocate energy preferentially to vital processes that promote immediate survival but at the expense of allocations to other demands (e.g., somatic growth or development of the immune system), ultimately limiting (constraining) the development of an optimal phenotype (*31*, *41*). The benefit of immediate survival in the face of adversity may ultimately be countered by the cost of increased risks of cardiometabolic disease, inflammatory disease, or other conditions that result from suboptimal developmental trajectories.

The PAR model emphasizes the possibility that uterine and early-life conditions provide accurate cues regarding the future environmental conditions the individual will be exposed to across their entire life (*31*, *42*). Functionally, PARs are assumed to anticipate future environmental conditions so that there is a stronger likelihood an individual’s phenotype will be suited to those conditions (*43*–*45*). The PAR model argues that nutritional deprivation experienced during pre- or postnatal developmental stages triggers predictive physiological responses that guide responses to stress events experienced later in life (*43*). These responses are thought to potentially make individuals more efficient in dealing with postnatal stressors due to heightened sensitivity. From an evolutionary standpoint, these responses are seen as advantageous until reproductive age and for reproduction; however, their effects in the postreproductive period, either beneficial or deleterious, are not relevant to an individual’s evolutionary fitness (*43*). But given that exposures to stressors can alter development in ways that increase one’s capacity to respond to those conditions (e.g., increased capacity to activate allostatic mechanisms), such predictive responses can potentially lead to relatively good health outcomes later in life if conditions remain relatively constant throughout life (*33*, *39*). However, health later in life might be compromised if prenatal and early-life conditions are not accurate predictions of environmental conditions later in life.

The allostatic load model recognizes the potential effects of the accumulation of stress exposures throughout and beyond development (*39*). Allostatic theory explains how physiological responses to environmental stressors maximize chances of survival and limit somatic damage. But over time, repeated allostatic activity (stress responses) leads to somatic damage and reduced resilience to further stressors (stress-related physiological dysregulation) (*39*). The accumulated damage produced by these repeated stress responses is called allostatic load (*46*). If allostatic responses are shaped by severe stressors early in life, this may lead to life-long dysregulation of stress responses. Such dysregulation may manifest as hyper- or hyporesponsiveness of the HPA axis and other systems, reduced resilience, and impairment of allostatic systems (i.e., reduced ability to return to physiological baselines and to limit damage upon exposure to stressors and reduced capacity for recovery) (*39*). Low birth weight (a commonly used indicator of the intrauterine environment in the DOHaD literature) and other indicators of adverse prenatal and early-life conditions are significantly associated with higher allostatic loads in adulthood (*39*, *47*). Numerous studies have found associations between higher allostatic load and poor outcomes in adulthood, such as an increased risk of all-cause mortality (*48*–*50*), Alzheimer’s disease (*51*), cardiovascular disease (*52*), cancer (*53*), and diabetes (*54*).

### Previous studies of malnutrition and the DOHaD

Several studies have examined the possible effects on adult health outcomes of malnutrition experienced in utero or in early childhood during historical famine events and in the context of seasonal malnutrition. For example, Cheng *et al.* (*55*) found that individuals with prenatal or early-life exposure to the Great Chinese famine (1958–1962) experienced significantly higher rates of tuberculosis compared to unexposed individuals. Moore *et al.* (*56*) found that individuals born during the “hungry season” in 20th-century Gambia were more susceptible to infectious disease and had higher hazards of death as adults compared to those who were born during the “harvest season.” These cases suggest that, as mentioned above, malnutrition early in life, when many components of the immune system undergo growth and development, can have deleterious effects on long-term immune function (*56*), resulting in elevated risks of morbidity and mortality with respect to infectious causes throughout life.

There are numerous studies demonstrating links between early-life malnutrition and poor outcomes from noncommunicable diseases in adulthood. For example, Luo and Kuo (*57*) found that fetal exposure to the Great Chinese Famine was associated with a higher risk of adult-onset diabetes, and Lumey *et al.* (*58*) found that individuals exposed during early gestation to the Ukrainian Holodomor Famine of 1932–1933 had more than twice the risk of type II diabetes in adulthood compared to unexposed people. Studies of the Dutch Famine (also called the Dutch Hunger Winter, approximately 1944–1945) have found similar associations, e.g., higher risks of coronary heart disease, shorter life spans, a faster pace of biological aging, and other negative outcomes in adulthood for exposed versus unexposed individuals (*59*–*61*). Further, studies of the Dutch Famine have highlighted the importance of the timing of exposures, as outcomes have varied depending on an individual’s gestational age during exposure to the famine event (*36*, *61*, *62*). Using cohorts from preindustrial Finland, Hayward and colleagues (*63*) explicitly tested the PAR hypothesis by evaluating survival rates and fertility of individuals during the 1866–1868 famine who experienced differential crop yields at birth. Contrary to PAR predictions, individuals born during higher crop yields displayed higher famine survival, and for males only, the interaction of social class was also associated with this survivorship, so that rich and middle-class males displayed higher survivorship regardless of birth-year crop yields (*63*). Some studies have examined the associations between historical famine events and mortality in general. Klemp and Weisdorf (*64*) found substantial differences in risk of death at age 10 and survivorship between English children born during a famine (approximately 1727–1728) and those born 5 years later. However, Kannisto and colleagues (*65*) and Song (*66*) found that mortality in later life was not higher for famine birth cohorts in 19th-century Finland and 20th-century China, respectively. These variable findings highlight the importance of understanding context and the mechanisms underlying DOHaD.

Similar analyses, linking direct evidence of early-life nutritional stress (plausibly during times of famine) with later life outcomes at the individual or cohort level, have not yet been published for the medieval period, in part because the relevant historical data are not available. For this study, the combination of incremental dentine analysis and survival/hazard analysis allows us to assess the association between survival of severe malnutrition early in life and morbidity and mortality later in life in medieval England and thus deepen the temporal scope of our understanding of the long-term consequences of famine and developmental stress more generally.

### Stable isotopic approaches to dietary and nutritional stress reconstructions in past populations

For this study, we examine patterns of developmental stress for *n* = 275 medieval individuals by focusing on stable isotopes informative about diet early in life. It is possible to estimate the diet of an individual by comparing the nitrogen stable isotope ratios (δ^15^N) and carbon stable isotope ratios (δ^13^C) of their skeletal tissues to those of potential food sources, such as contemporaneous animal remains. A rise in trophic level foodstuffs will result in δ^13^C and δ^15^N rising (covariance), so the tissues of the consumer of a plant-based diet will have lower δ^13^C and δ^15^N than an omnivore. The length of the food chain will also affect the number of trophic level shifts to the end consumer. By using historical data about diet, which may provide information about the inclusion of marine foods and the type of grains consumed, we can also infer the consumption of marine foods or grains that have different stable isotope values; for a discussion of this, see the study of Lee-Thorp (*67*). For example, consumers of marine foods tend to have higher δ^15^N values coupled with higher δ^13^C values than consumers of terrestrial foods, while consumers of plants using the C_4_ photosynthetic pathway, such as maize and millet, will have high δ^13^C values relative to consumers of C_3_ plants, such as wheat and barley (*67*).

When one’s diet does not provide sufficient energy and/or protein, the body can enter a catabolic state, in which body tissues such as muscle and fat are recycled to provide energy and the amino acids required to build new tissues. The δ^13^C and δ^15^N values of body tissues reflect not only the diet of an individual but have been observed to vary in rapidly growing tissues, such as hair and fingernails, during periods of nutritional stress when dietary intake is insufficient for the individual’s energy requirements (*68*, *69*). Neuberger *et al.* (*70*) report the segmental analysis of hair for forensic investigation of 15 adults, and a child believed to have died as the result of food deprivation. They found a trend for the δ^15^N values of the hair segments to rise as body mass index (BMI = weight in kilograms divided by the square of height in meters of the individual) decreased. This pattern is consistent with earlier studies of δ^15^N values of hair from individuals deprived of food (*69*, *71*). Neuberger *et al.* (*70*) also noticed that, among the adults, δ^13^C values decreased in phase with BMI. An observed drop in δ^13^C was thought to be the result of the breakdown of body fat deposits to maintain the energy requirements of the body (*72*). δ^13^C values of body fat deposits are ~3 per mil (‰) lower than other body tissues such as muscle (*70*, *73*). They may also be used in the synthesis of new body tissues in the absence of dietary carbohydrate by converting acetone derived from ketogenesis (derived from fats) into glucose (*74*). Unlike the adults in the study, Neuberger *et al.* (*70*) found the δ^13^C and δ^15^N values covaried in the same direction in hair from a 10-month-old child, suggesting a different metabolic pathway during food deprivation in a rapidly growing infant, possibly because the body tissues are being recycled not only to provide energy but also to synthesize new body proteins during growth. The observed reduction in δ^13^C in the tissues of nutritionally stressed adults complements the findings of Mekota *et al.* (*69*) who recorded a concurrent rise in δ^13^C and a fall in δ^15^N in the hair of patients recovering from anorexia nervosa once refeeding had started and those of Lehn *et al.* (*75*) in the identification of unknown individuals with a poor quality diet. Cherel *et al.* (*76*) examined the effects of fasting in king penguins on feather composition, attributing an increase in δ^15^N to the catabolism of lean tissues and a decrease in δ^13^C, linked to lipid content, as indicative of protein sparing and lipid use during fasting. Although energy partitioning (the relative proportion of energy derived from protein and fat stores) varies between individuals and is influenced by the prestarvation percentage of body fat to lean mass, both stores are used during prolonged fasting (*77*).

The δ^13^C and δ^15^N values in collagen from bone and dentine reflect the protein element of one’s diet during the formation of these tissues, with bone providing an average of the diet recorded during lifelong remodeling. However, primary dentine does not remodel, and as teeth form in a regular pattern, this means that we can produce an approximate temporal sequence of diet throughout the formation of a tooth (*78*–*80*). Several researchers have developed methods for sampling in the direction of growth to establish a timeline and refine the approximate age assigned to each tooth and/or section of a tooth [e.g., (*78*, *81*–*85*)]. Permanent human teeth, apart from the third molar, have been shown to develop during childhood and adolescence at the same time for all individuals, regardless of age, sex, period, or nutritional status, within an error of about ±6 months (*86*–*88*). Hence, it is possible to compare the diet of different individuals at approximately the same age. It is important to note that the horizontal sampling technique used in the method of incremental dentine collagen measurement will inevitably cause an averaging of the signal observed: Each sample represents a rolling average, and this will result in an attenuated signal over an apparently longer period of time (*89*). Refinements of the method have improved the temporal resolution, but a degree of averaging still persists (*83*, *84*). The temporal resolution, however, is still much greater than that obtained by bulk sampling of skeletal elements, which form at different times of life or remodel at differing rates.

It has been shown that the stable isotope ratios in dentine collagen can also provide sufficient temporal resolution to record short-term changes in nutritional status: Where there is historical evidence for a short-term change due to a known event, such as a child entering a monastic order or migrating between two areas with differing dietary behavior, this can be matched to observed isotopic changes (*78*, *90*, *91*). The effect on stable isotope ratios in dentine collagen can show the same pattern observed in the modern hair and fingernail studies discussed above, particularly δ^15^N values, which can rise during a famine period, and δ^13^C values, which may decrease if fat stores are used. For example, in the teeth of children who died during the Great Irish Famine, the dentine profiles showed a rise in δ^15^N values corresponding with stable or decreasing δ^13^C values. This pattern occurs just before the introduction of maize as a famine relief food, after which the δ^15^N values returned to a lower value, while the δ^13^C values reflected the consumption of corn, a C_4_ plant, as a major element in the diet (*92*). The patterned change in stable isotope ratios during this period of nutritional distress was described as “opposing covariance” and has been observed in dentine collagen stable isotope ratio profiles in this and other populations as evidence of short-term nutritional distress [(*92*), e.g., (*93*–*96*)]. The presence or absence of this characteristic pattern of rising δ^15^N with either flat or opposing covariance of δ^13^C in a dentine collagen profile can therefore be used as a biomarker in evaluating the exposure of an individual to nutritional stress during the formation of a tooth. While there are some conditions when this pattern may occur, such as a dietary shift from a plant-based C_4_ diet to an omnivorous C_3_ diet, there is no evidence that C_4_ plants were available or widely consumed in Britain during the period of this study.

**Fig. 2.**
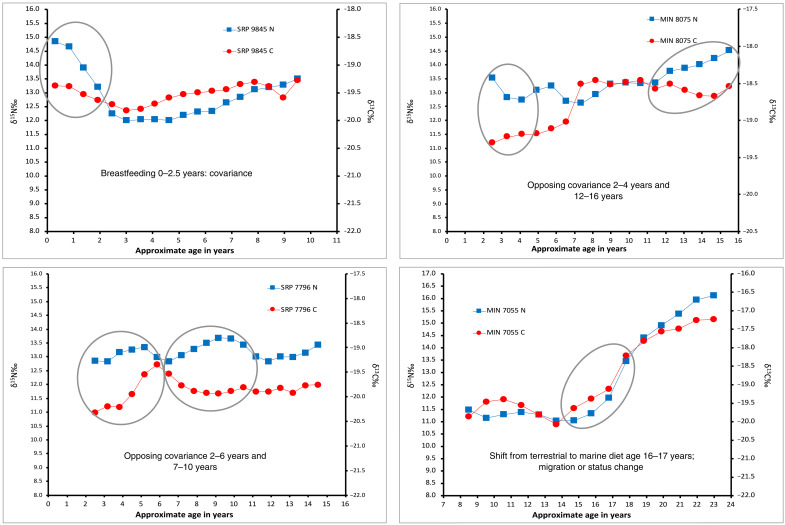
δ^15^N and δ^13^C dentine collagen profiles. These show patterns consistent with breastfeeding (covariance), nutritional stress (opposing covariance), and migration or status change (dietary shift terrestrial to marine) selected from this study. Axes have been adjusted to show these changes more clearly.

We use patterns of opposing covariance of δ^15^N and δ^13^C in dentine profiles (see examples in Fig. 2) to identify which individuals included in our study experienced severe childhood nutritional stress (i.e., developmental stress), and we compare demographic and pathological patterns among those with and without such signatures to evaluate the possible developmental origins of health in medieval England and which of the models of DOHaD described above might be most relevant for this context.

### Skeletal biomarker of inflammation

To examine the possible role of inflammatory responses in linking developmental stress to health outcomes later in life, we examine patterns of periosteal new bone formation (PNBF) for *n* = 144 individuals for whom we have δ^13^C and δ^15^N data. Trauma to or infection and inflammation of the periosteum (the osteogenic connective tissue that covers most of the surfaces of bones) can stimulate the production of abnormal new bone (PNBF). Because periosteal new bone can be caused by a variety of factors that may not be identifiable in bioarchaeological contexts, PNBF is often used as an indicator of exposure to stressors of unknown etiology (i.e., a nonspecific stress indicator) in bioarchaeological research (*97*). PNBF can reflect episodic or cumulative exposures to stressors across the life span. Previous work in the context of medieval London has found that PNBF, at least in the active (unhealed) state at the time of death, is associated with relatively low survivorship and high hazards of death and thus may be a good indicator of frailty (*98*, *99*). Further, it has also been found in the context of medieval London to be significantly associated with periodontal disease, when controlling for age, which suggests that PNBF may be reflective of an underlying hyperinflammatory status (*100*).

### Study aims

Our goals in this study are to examine the associations between stable isotopic indicators of severe childhood nutritional stress and (i) survivorship, (ii) hazards of death, and (iii) a skeletal biomarker of inflammation (PNBF) to determine whether the experience of nutritional stress (perhaps during times of famine) substantially affected health outcomes later in life during the medieval period in England. We hypothesized that individuals who experienced severe nutritional stress during childhood (as indicated by patterns of opposing covariance in δ^15^N and δ^13^C values in incremental dentine profiles) will exhibit poorer health outcomes (elevated hazards of death, reduced survivorship, and higher rates of PNBF) compared to those without evidence of severe childhood nutritional stress. Further, given our larger goal of understanding the possible mechanisms underlying previously observed trends in health before and after the Black Death, we examine temporal trends in the prevalence of individuals with stable isotopic signatures of severe childhood nutritional stress across the medieval period. Previous research found that frequencies of linear enamel hypoplasia (LEH) increased and survivorship decreased in the 13th century CE (late pre–Black Death period) compared to the 11th to 12th centuries CE (early pre–Black Death period) and that LEH decreased and survivorship increased in the post–Black Death period (approximately 1350 to 1540 CE) (*3*). Thus, we hypothesized that the prevalence of severe nutritional stress was highest in the late pre–Black Death period and decreased after the epidemic.

## RESULTS

### Incremental dentine profiles

Figure 2 shows examples of dentine profiles generated from individuals included in our study, including profiles showing opposing covariance of δ13C and δ15N that indicate distinct, visible periods of nutritional stress before an individual transitioned to a stable state. Additional patterns were noted where nutritional stress at the beginning of a tooth profile appeared to be resolving, with δ15N decreasing from a high value as δ13C increased, and at the end of a profile, where a rising δ15N and flat or declining δ13C suggest the onset of nutritional distress that had not yet peaked or resolved. It is important to note that breastfeeding and weaning can produce a pattern in an early-forming part of a tooth whereby δ^15^N and δ^13^C values concurrently decrease, and this pattern can thus be distinguished from the nutritional stress pattern (Fig. 2). Other life history details visible in dentine profiles include major dietary shifts, such as a major shift from terrestrial to marine resources, suggesting a change in status or migration. Table 1 displays the distribution of individuals that we identified as having or lacking isotopic signatures of severe childhood nutritional stress by time period and age group.

**Table 1.** Distribution of childhood nutritional stress by age group and time period. Note that for this table, the “general medieval (1000 to 1540 CE)” time period includes only those individuals who could not be assigned to any of the other specific time periods (early pre–Black Death, late pre–Black Death, circa–Black Death, or post–Black Death).

Time period	Age group	No nutritional stress	Nutritional stress
General medieval (1000 to 1540 CE)	**<30**	17	15
	**30+**	7	11
	**Unknown**	1	0
Early pre–Black Death (1000 to 1200 CE)	**<30**	8	2
	**30+**	4	3
	**Unknown**	2	1
Late pre–Black Death (1200 to 1250 CE)	**<30**	11	23
	**30+**	7	5
Circa–Black Death (1250 to 1350 CE)	**<30**	40	14
	**30+**	10	13
	**Unknown**	1	0
Post–Black Death (1350 to 1540 CE)	**<30**	28	21
	**30+**	15	14
	**Unknown**	1	1

### Survival and hazard analysis

**Fig. 3.**
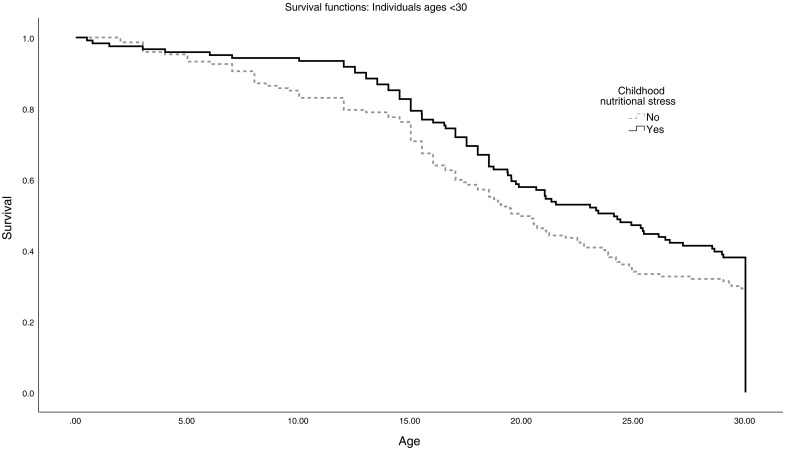
Kaplan-Meier survival curves for individuals under the age of 30.

**Fig. 4.**
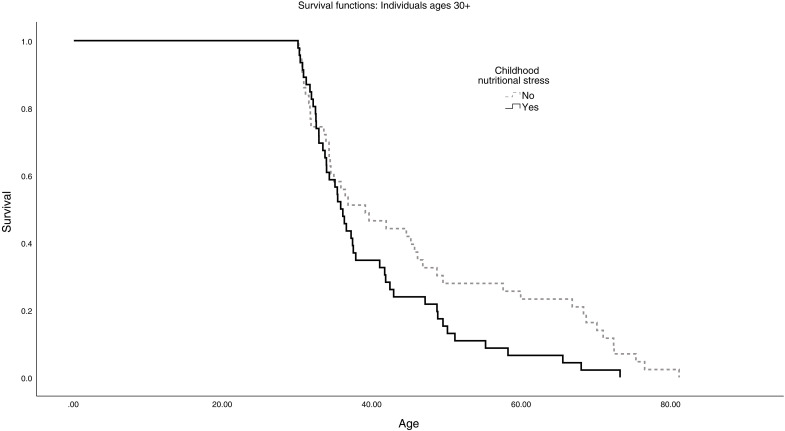
Kaplan-Meier survival curves for individuals aged 30 and older.

Across the medieval period (approximately 1000 to 1540 CE; *n* = 268 individuals with age estimates), when we restrict analyses to those people who died at ages younger than 30 years (see Materials and Methods for the rationale for performing analyses separately for those above and below the age of 30), we find significantly higher survivorship for those with signatures of severe childhood nutritional stress but no significant difference in hazards of death between those with and without signatures of severe childhood nutritional stress (Fig. 3 and Tables 2 and 3). Conversely, when we consider only those individuals who died at ages 30 years and older, we find significantly higher survivorship (Fig. 4) and significantly lower hazards of death for people without signatures of severe childhood nutritional stress compared to those with such signatures (Tables 2 and 3).

**Table 2.** Results of Kaplan-Meier survival analyses. Mean survival times (mean ages at death) in years are shown with 95% confidence intervals in parentheses. Note that for this table, the “general medieval (1000 to 1540 CE)” time period includes all individuals assigned to the other specific time periods (early pre–Black Death, late pre–Black Death, circa–Black Death, or post–Black Death) and those who could not be assigned to any of the other specific time periods.

Time period	Age group	No nutritional stress	Nutritional stress
General medieval (1000 to 1540 CE; *n* = 268)	**<30**	20.10	22.32*
		(18.71–21.49)	(20.90–23.73)
	30+	45.89	39.85**
		(41.0–50.77)	(36.81–42.89)
Early pre–Black Death (1000 to 1200 CE; *n* = 17)	**<30**	22.66	26.95
		(19.20–26.13)	(23.27–30.64)
	30+	37.64	36.97
		(32.28–43.00)	(24.19–49.74)
Late pre–Black Death (1200 to 1250 CE; *n* = 46)	**<30**	23.97	20.94
		(21.18–26.75)	(18.80–23.09)
	30+	37.35	33.29
		(29.11–45.58)	(31.76–34.82)
Circa–Black Death (1250 to 1350 CE; *n* = 77)	**<30**	18.50	23.13**
		(16.04–20.98)	(19.77–26.50)
	30+	48.97	40.23
		(37.13–60.81)	(34.99–45.47)
Post–Black death (1350 to 1540 CE; *n* = 78)	**<30**	22.13	23.53
		(20.05–24.22)	(21.44–25.62)
	30+	48.10	38.43*
		(38.65–57.55)	(33.26–43.59)

*P < 0.10.

**P < 0.05.

**Table 3.** Results of Cox proportional hazard analyses. The covariate effect is nutritional stress. Note that for this table, the “general medieval (1000 to 1540 CE)” time period includes all individuals assigned to the specific time periods (early pre–Black Death, late pre–Black Death, circa–Black Death, or post–Black Death) and those who could not be assigned to any of the other specific time periods. CI, confidence interval.

Time period	Age group	Exp(β) (95% CI)
General medieval (1000 to 1540 CE)	**<30**	0.83
		(0.65–1.06)
	**30+**	1.57*
		(1.01–2.44)
Early pre–Black Death (1000 to 1200 CE)	**<30**	0.64
		(0.23–1.84)
	**30+**	0.97
		(0.17–5.48)
Late pre–Black Death (1200 to 1250 CE)	**<30**	1.52
		(0.84–2.76)
	**30+**	1.06
		(0.30–3.73)
Circa–Black Death (1250 to 1350 CE)	**<30**	0.61*
		(0.38–0.98)
	**30+**	2.01
		(0.77–5.23)
Post–Black Death (1350 to 1540 CE)	**<30**	0.88
		(0.56–1.37)
	**30+**	2.20**
		(0.97–5.01)

*P < 0.05.

**P < 0.10.

As detailed below, circa–Black Death (approximately 1250 to 1350 CE; *n* = 78) and post–Black Death individuals (approximately 1350 to 1540 CE; *n* = 80) are overrepresented in our study. Thus, we also analyzed patterns of survivorship and hazards of death within each time period (early pre–Black Death, late pre–Black Death, circa–Black Death, and post–Black Death) to determine whether there was any temporal variation in the relationships between nutritional stress and health and thus whether the overall observed medieval pattern is potentially driven by conditions at the time of the Black Death or in the post–Black Death period. Survival analysis of individuals who died at ages younger than 30 reveals patterns that are generally consistent with our findings for the larger, pooled medieval time period sample. Specifically, individuals with signatures of severe childhood nutritional stress have higher estimated survivorship compared to those without such signatures for all periods except the late pre–Black Death period (Table 2). However, this finding is significant only for the circa–Black Death period. Survival analyses of individuals who died at ages 30 and older reveal higher survivorship for those without signatures of nutritional stress in each time period (consistent with our previous findings for the pooled medieval time period sample), but none of these findings are significant (Table 2). Cox hazard analyses of those people who died below the age of 30 are consistent with the survival analyses, i.e., generally indicating lower hazards of death for those with signatures of nutritional stress during all time periods, except the late pre–Black Death period, with the results significant only for the circa–Black Death period (Table 3). Cox hazard analyses of individuals who died at age 30 or above reveal lower hazards of death for individuals without nutritional stress for all periods except the early pre–Black Death period, with the results significant only for the post–Black Death period. In general, the results of these time period–specific analyses are consistent with those for the general medieval period analyses and suggest that the trends we observe across the entire medieval period are not biased by a greater representation of individuals from the circa– or post–Black Death period.

### Periosteal new bone formation

We find that PNBF is more common among individuals with signatures of nutritional stress compared to those without such signatures when we pool data across all ages and when we perform analyses separately for those people who died at ages younger than 30 years and those 30 years of age and older (Table 4). However, the findings were significant only for analyses including individuals of all ages and just those individuals aged 30 years and older.

**Table 4.** Prevalence of PNBF among people with and without signatures of childhood nutritional stress. Note the “all ages” group include two individuals for whom age estimation was not possible and who are thus not included in the <30 or 30+ groups.

Age group	Nutritional stress	PNBF absent	PNBF present	χ^2^
All ages	**No**	49	24	5.46*
		(67.1%)	(32.9%)	
	Yes	34	37	
		(47.9%)	(52.1%)	
<30	**No**	36	14	1.20
		(72.0%)	(28.0%)	
	Yes	27	17	
		(61.4%)	(38.6%)	
30+	**No**	12	10	3.80**
		(54.5%)	(45.5%)	
	Yes	7	19	
		(26.9%)	(73.1%)	

*P < 0.05.

**P < 0.10.

### Temporal trends in nutritional stress

We find significant variation in the prevalence of individuals with signatures of childhood nutritional stress across the four time periods included in our study (Table 5). Notably, and supporting our original hypothesis, there was an increase in the prevalence of individuals with signatures of nutritional stress in the late pre–Black Death period compared to the early pre–Black Death period and a decrease after the Black Death.

**Table 5.** Prevalence of childhood nutritional stress over time. Note that this table excludes individuals who could not be assigned to a specific time period within the general medieval period.

Time period	No nutritional stress	Nutritional stress	χ^2^
Early pre–Black Death (1000 to 1200 CE)	14	6	9.72*
	(70.0%)	(30.0%)	
Late pre–Black Death (1200 to 1250 CE)	18	28	
	(39.1%)	(60.9%)	
Circa–Black Death (1250 to 1350 CE)	51	27	
	(65.4%)	(34.6%)	
Post–Black Death (1350 to 540 CE)	44	36	
	(55.0%)	(45.0%)	

*P < 0.05.

## DISCUSSION

### Developmental stress and health outcomes

The significantly higher survivorship for individuals with signatures of childhood nutritional stress who died below the age of 30 compared to those without such signatures might indicate that those people who experienced but survived such stress were a particularly resilient group of individuals with respect to the causes of death that are most common during childhood, adolescence, and young adulthood. Geber (*101*) similarly suggested that the higher mean-age-at-death for non-adults with dental enamel defects (reflecting early-life stress) compared to those without them in the Kilkenny workhouse cemetery from Ireland (approximately 1847–1851) might indicate that children and adolescents who had experienced previous stress were better able to subsequently cope with the stressors associated with the Great Irish Famine (approximately 1845–1862).

However, in our study, the lack of a significant difference in hazards of death for those who died below the age of 30 with and without signatures of childhood nutritional stress cautions us against overinterpreting the survival analysis findings for this age group. On the basis of the δ^13^C and δ^15^N profiles for the individuals in our study, it is possible for us to identify people who likely died as a result of nutritional stress in childhood. That is, as detailed above and shown in Fig. 2, for some individuals in our study, the period of nutritional stress (as indicated by a rise in δ^15^N while δ^13^C is flat or falling) had not peaked or resolved at the time of their deaths. In other cases, the nutritional stress had resolved just before the estimated age at death, which might suggest that severe malnutrition was ultimately a contributing factor in the deaths of those individuals. However, it is possible that some children included in our study died as a result of malnutrition but are not identified as such; that is, some individuals may have died from severe malnutrition in childhood before they manifested a pattern of opposing covariance pattern and would thus be scored in our study as not having signatures of nutritional stress. This might affect our survivorship and hazard findings for individuals who died below the age of 30 (i.e., the subgroup in our analyses that includes those who died in childhood), such as biasing the estimated survivorship for those without signatures of nutritional stress downward or masking differences in hazards between those with and without signatures of nutritional stress. Given that the rate of false negatives for “nutritional stress” is unknown in our study, the results for individuals who died below the age of 30 should be interpreted with caution.

The significantly lower survivorship and significantly higher hazards of death for individuals with signatures of childhood nutritional stress who died above the age of 30 compared to those without them might be evidence of a DOHaD effect in medieval England. Processes that promoted survival during childhood in the face of severe nutritional stress may have had effects on development that were beneficial for survival up through young adulthood (i.e., before age 30). But these developmental adaptations may have ultimately increased the chances of poor health and mortality outcomes later in middle and late adulthood. Similar results have been reported recently with respect to different measures of developmental stress: LEH and fluctuating asymmetry (FA). Wyatt and colleagues (*102*) found in the context of medieval Ireland that the presence of LEH was associated with higher survivorship when analyses were restricted to individuals who died below the age of 18, but it was associated with lower survivorship when analyses included individuals of all ages. Wigley and colleagues (*103*) report that for medieval and postmedieval individuals from Northern England, higher FA was significantly positively associated with age-at-death among immature individuals but negatively associated with age among mature individuals.

Our findings may be consistent with the allostatic load model and reflect both (i) developmental adaptations that set the stage for dysregulation of stress responses and (ii) the accumulation of damage via exposure to repeated stressors. That is, individuals who experienced childhood nutritional stress (and were able to survive past the period of malnutrition) and continued to experience environmental stressors across their lives may have ultimately been at relatively high risks of death from causes common in middle and late adulthood. These findings may also suggest that, in this population, there was a mismatch between the prenatal/early-life and later-life environments. That is, as expected under the PAR model, disease in later adulthood may have been the outcome of early environments providing an inaccurate forecast of environmental conditions in adulthood (*31*). Perhaps the people in our study who suffered early-life nutritional stress underwent developmental adaptations as a result that shaped their physiology for a future of nutritional deprivations, but they subsequently experienced relative nutritional abundance in adulthood. Thus, they may have suffered higher rates of diseases common in later adulthood, such as cardiovascular disease and other “degenerative” diseases, as has been suggested to explain the relationships between early-life stress and noncommunicable disease outcomes for present-day people.

Given, as noted above, the possible association between PNBF and hyperinflammation, our findings of higher frequencies of PNBF among people with signatures of childhood nutritional stress (significantly so for adults who died at ages above 30) might reflect heightened inflammatory responses among these individuals that were programmed during early development in response to malnutrition. Such heightened inflammatory responses, in addition to making individuals more likely to produce PNBF, could also have contributed to higher risks of death in middle and late adulthood. That is, inflammation might have been the mechanism underlying the associations between childhood nutritional stress and both PNBF and higher hazards of death after age 30 in medieval England. Wigley and colleagues (*103*) suggest such a mechanism might explain the association they find between FA and PNBF in the context of medieval and postmedieval Northern England.

There is evidence from living people to suggest that inflammation is a plausible mechanism linking early-life stress and adult health outcomes (*104*). For example, deRosset and Strutz (*105*) found that birth weight (an indicator of intrauterine environment) is significantly, inversely associated with C-reactive protein concentration (a marker of chronic inflammation) in adulthood. Further, inflammatory responses have been found to contribute to higher risks of cardiovascular disease, metabolic syndrome, cancer, and other conditions in adulthood (*106*–*110*). It is also possible that the association between nutritional stress and PNBF that we find may reflect the negative effects of malnutrition on the development of the immune system, which made the individuals in our study less able to clear infection with pathogens capable of stimulating the formation of PNBF [see also (*103*)].

Given that we have some evidence that individuals who survived severe nutritional stress subsequently experienced better health outcomes up until the age of 30, we do not consider our findings to strongly support the developmental constraints model. Under that model, we would expect to find less of a prolonged survival advantage for those exposed to nutritional stress compared to those unexposed in general. However, as noted above, given the lack of significant findings from the hazard analyses for this age group, we are viewing these patterns for individuals below the age of 30 with caution. Future analyses with larger sample sizes may allow for the integration of data on age at exposure to nutritional stress and thus timing of the window between stress exposure and death, which would allow for better testing of the developmental constraints model.

### Temporal trends in medieval nutritional stress

The significant increase in the prevalence of individuals with signatures of nutritional stress in the late pre–Black Death period (1200 to 1250 CE) versus the early pre–Black Death period (1000 to 1200 CE) and a significant decrease in nutritional stress after the Black Death (1350 to 1540 CE) aligns with previous findings with respect to skeletal biomarkers in medieval London. Specifically, previous work focusing on some of the same cemeteries used in the current study produced evidence that the prevalence of LEH on the mandibular canine increased, and stature (at least for estimated males) decreased in the late pre–Black Death period compared to the early pre–Black Death period (*3*). Following the Black Death, frequencies of LEH decreased, and male stature increased (*3*). LEH and achieved adult stature both reflect exposure to developmental stressors of various kinds, including but not limited to nutritional stress. The findings of our current study suggest that, at least for some of those individuals, the specific stressor was malnutrition during development. Given the association between nutritional status and immunity and health established for recent and present-day populations [e.g., (*34*, *111*)], our findings are also consistent with previous evidence of declining survivorship (and, by inference, health) before the Black Death and improvements in its aftermath in London and Denmark (*1*, *2*, *112*).

It is important to note that the temporal trends in the prevalence of individuals with signatures of childhood nutritional stress do not appear to be the result of biases in the sampling of catastrophic versus attritional burials for our study (see Materials and Methods below for further details about the nature of these burials). As shown in Table 6, there is variation in the proportions of each burial type across the four time periods examined in this study. However, compared to the other time periods, we included the lowest proportion of catastrophic (type D) burials in the sample from the late pre–Black Death period. This is the same period for which we observe the highest prevalence of people with signatures of childhood nutritional stress. If our findings with respect to temporal trends in nutritional status were an artifact of temporal variation in burial types (among the individuals included in this study), we would expect the opposite finding, i.e., the lowest prevalence of individuals with signatures of severe nutritional stress would be found for the late pre–Black Death period, which had the lowest proportion of individuals from putative famine burials. The fact that the fewest catastrophic burials in our study date to the late pre–Black Death period might suggest that the prevalence of nutritional stress actually increased even more markedly in the late pre–Black Death period than we observe here.

**Table 6.** Number of individuals from each cemetery by time period and burial type. Note that for this table, the “general medieval (1000 to 1540 CE)” time period includes all individuals who could not be assigned to any of the other specific time periods (early pre–Black Death, late pre–Black Death, circa–Black Death, or post–Black Death).

Time period	Cemetery	Attritional burial	Plague/mass burial	Total
Early pre–Black Death (1000 to 1200 CE)	St. Mary Spital	6	4	20
	St. Nicholas Shambles	10	0	
**Late pre**–**Black Death (1200 to 1250 CE)**	St. Mary Spital	42	4	46
Circa–Black Death (1250 to 1350 CE)	East Smithfield	0	42	78
	St. Mary Spital	5	4	
	Thornton Abbey	0	27	
Post–Black Death (1350 to 540 CE)	St. Mary Graces	11	16	80
	St. Mary Spital	50	3	
General medieval (1000 to 1540 CE)	St. Mary Spital	1	0	51
	Thornton Abbey	50	0	

As detailed below in Materials and Methods and in the supplementary data file (table S1), we have not sampled the same teeth from all individuals included in our study, and we acknowledge that stressors at some ages will therefore not be equally captured for all individuals included in our study. That is, there may be people in our study who are scored as not having experienced severe childhood nutritional stress but who might have experienced such stressors before or following the development of the teeth we examined. We expect that this would tend to minimize observable differences in survivorship, hazards of death, and frequencies of PNBF between those with and without observable signatures of nutritional stress—i.e., we expect that the differences we observe are perhaps an underestimate of true differences in health outcomes between individuals who experienced severe childhood nutritional stress and those who did not.

Ultimately, the results of our study suggest that early-life nutritional stress was associated with poor health outcomes in middle and later adulthood in medieval England in the context of plague and under normal conditions of mortality. These findings suggest that the relationship between intrauterine/early-life malnutrition and increased risks of poor health conditions in adulthood that has been observed in present-day populations also existed in the medieval period in southern England. Further, given that our findings suggest that the negative effects of early-life stress did not manifest until middle and later adulthood and given observations in present-day populations of a positive correlation between noncommunicable diseases and age, it is possible that the conditions people suffered from at those ages in medieval England included or were even predominantly noncommunicable diseases such as cardiovascular disease and cancer.

Without knowing the causes of death for the individuals in the nonplague burials or the specific adaptations that occurred during development in response to malnutrition among the people included in our study, it is difficult (if not impossible) to discern which model regarding DOHaD best aligns with the patterns we observe here. However, future research integrating epigenetic evidence could provide further resolution. As mentioned above, several studies have found epigenetic modifications associated with developmental stress and health outcomes in adulthood, for example, mechanistically linking prenatal or early-life exposure to nutritional stress and increased risk of chronic diseases in adulthood [e.g., (*36*–*38*)]. While bioarchaeological epigenetics is in its infancy, it might be possible in future work to examine epigenetic differences between medieval people with and without signatures of nutritional stress. In addition, emerging work on the microbiome in past populations may help clarify the mechanisms at play in this population (*113*). Evidence from living people indicates that prenatal/early-life nutritional status affects the microbiome [e.g., (*114*, *115*)] and that the microbiome influences adult health (*116*, *117*). Gancz and colleagues (*118*) recently reported on ancient oral microbiomes from Great Britain (including individuals from medieval London), and their findings suggest associations between microbial communities and systemic diseases such as periostitis and joint pathologies. Last, approaches linking immunology and bioarchaeology (osteoimmunology) are laying the groundwork for the assessment of inflammatory phenotypes in past populations (*119*, *120*), which could potentially be informative regarding whether inflammatory responses linked early-life nutritional stress and adult health outcomes in medieval England or other contexts of interest.

## MATERIALS AND METHODS

### Cemeteries

We included in this study individuals from a rural cemetery in Lincolnshire (Thornton Abbey) and several cemeteries in London (East Smithfield, St. Mary Graces, St. Mary Spital, and St. Nicholas Shambles). We only included individuals from each cemetery for whom we could obtain teeth for dietary stable isotopic analyses. The individuals included in this study (*n* = 275) represent all ages and sexes and both higher and lower socioeconomic strata. As detailed below, some individuals represent normal (attritional) causes of mortality, while others were victims of catastrophic mortality (famine or plague). Most of the individuals (*n* = 224 of 275) included in this study can be placed into one of four periods within the general medieval period (approximately 1000 to 1540 CE): 1000 to 1200 CE (early pre–Black Death), 1200 to 1250 CE (late pre–Black Death, 1250 to 1350 CE (mostly from 1348 to 1350; circa–Black Death), and 1350 to 1540 CE (post–Black Death). The numbers of individuals from each cemetery, time period, and burial type (attritional or catastrophic) are shown in Table 6.

St. Nicholas Shambles was a small parish church in London, established in 1144 or 1187 CE and closed in 1548 to 51 CE (*121*). The associated burials date to the 11th to 12th centuries CE and, on the basis of archaeological evidence, represent attritional mortality (*n* = 10 included in this study). St. Mary Spital priory and hospital were founded in 1197 CE and in use until 1539 CE. Burials in the main cemetery associated with the priory and hospital have been chronologically divided, based on radiometric dating, into four phases: period 14 (approximately 1120 to 1200 CE), period 15 (approximately 1200 to 1250 CE), period 16 (approximately 1250 to 1400 CE); and period 17 (approximately 1400 to 1539 CE) (*122*, *123*). Further, within each period, there are several burial types based on the number and arrangement of the interred individuals: single interments (type A), small group burials (types B and C), and larger group burials (type D). The type D burials are considered to represent famine-related catastrophic mortality, and types A, B, and C represent attritional mortality (*122*). While the type B and C burials have been considered attritional burials, we acknowledge that there may be questions about how certain we can be that these small group burials truly represent normal mortality patterns. Therefore, for the original sampling strategy for our study, we took a conversative approach and excluded individuals in type B and C burials so that, in future work, we can compare individuals most likely to represent patterns of normal mortality (type A burials) versus crises mortality (type D burials). For this study, we include individuals (*n* = 119) from all time periods in St. Mary Spital and from type A (*n* = 104) and type D (*n* = 15) burials.

The East Smithfield Black Death Cemetery (approximately 1348 to 1350 CE) was established in 1348 CE in advance of the anticipated arrival of the plague pandemic, and because it was only used for the duration of the Black Death in London (*124*), most if not all people interred there died of plague, as supported by ancient DNA analyses. We include *n* = 42 people from East Smithfield in this study (*125*). The Abbey of St. Mary Graces (approximately 1350 to 1538) was established after the Black Death ended in 1350 CE, and the associated cemetery was in use until 1538 CE (*124*, *126*). In addition to noncatastrophic burials of lay people, wealthy benefactors, and monks, included in the St. Mary Graces cemetery are victims of 14th-century CE plague (the plague of 1361 CE or a subsequent outbreak), as indicated by their location in an area spatially distinct from the rest of the burials and ancient DNA evidence of the plague bacterium *Yersinia pestis* (*127*). We include 27 individuals, representing plague (*n* = 11) and attritional mortality (*n* = 16), from St. Mary Graces in our study.

Thornton Abbey was established in Lincolnshire in 1139 CE and in use until 1539 CE. Recent excavations revealed a cemetery for the associated St. James hospital, which includes attritional burials and a mass burial (*128*). The mass burial is radiometrically dated to the 14th-century CE, and ancient DNA evidence of *Y. pestis* indicates that individuals in the mass burials are Black Death victims (*n* = 27 included in this study) (*129*). The attritional burials have not been phased into specific pre– or post–Black Death periods, and thus we use them here (*n* = 50) to represent normal morality across the general medieval period approximately 1139 to 1539 CE.

### Age estimation

None of the individuals included in this study have any associated identifying information from headstones, burial records, or other sources, so we estimated the ages-at-death for all individuals included in this study from skeletal features. To estimate ages-at-death for fully mature individuals (those with fused epiphyses) in this study, we used the method of transition analysis as described by Boldsen and colleagues (*130*). This Bayesian approach to age estimation, in general, yields point estimates of age, even for the oldest adults, and avoids the limitation of age mimicry (estimates that are biased toward known-age references samples) that is associated with conventional methods of estimation. Following Boldsen *et al.* (*130*), ages for adults in this study were estimated on the basis of features of the pubic symphysis, iliac auricular surface, and cranial sutures that change in predictable ways with senescence. This method, like all methods of age estimation based on macroscopic changes to the skeleton, has limitations with respect to accuracy and precision. However, for this study, we are focused on aggregate patterns, and the application of the same age estimation method to all adults included in our study mitigates concerns about the accuracy of individual age estimates. We estimated adult ages using the ADBOU Age Estimation software (originally provided by the Anthropology Unit in the Department of Forensic Medicine, University of Southern Denmark) and specified the informative “archaeological” prior distribution. This prior is based on data from 17th-century Danish rural parish records and thus represents a preindustrial mortality curve that is appropriate for analyses of medieval individuals (*131*).

To estimate ages for non-adult individuals, we used conventional methods based on dental development and eruption, as well as epiphyseal fusion (*132*–*137*). Across the entire general medieval period (approximately 1000 to 1540 CE), we have age estimates for a subsample of *n* = 268 individuals of the 275 total for whom we produced δ^13^C and δ^15^N profiles and identified signatures of nutritional stress, as detailed below.

### Incremental dentine analysis

We obtained teeth from 275 individuals for the isotopic analyses for this study with the permission of and following the ethical guidelines of the London Museum and the University of Sheffield. For most of the individuals included in our study, we sampled a single permanent tooth, but for some individuals from Thornton Abbey, we were able to sample a deciduous and a permanent tooth (details about the teeth sampled from all individuals included in this study are provided in table S1). In general, our goal was to sample teeth that provide information across a relatively long developmental window, and our sample of permanent teeth is exclusively canines, premolars, and molars. We avoided teeth with pathologies (caries and abscesses), extensive wear, and intact calculus to avoid interfering with future research focusing on those variables, and we selected teeth that were easily removed from the alveolar bone to avoid damage to the bone. Further, sampling for this study was in some cases opportunistic: To minimize destructive sampling in accordance with guidance from the London Museum, for many individuals in this study, we used teeth that had been previously sampled for paleogenomic analyses. We discuss the potential impact of not using the same tooth type across all individuals in this study in Discussion.

All dentine samples were prepared and measured at the Stable Isotope Laboratory, University of Bradford, England. The samples were demineralized in a 0.5 M aqueous solution of hydrochloric acid in a refrigerator at 4°C following the modified Longin method (*138*). The permanent tooth samples were cut into 1-mm-horizontal sections using a scalpel (*78*). The deciduous teeth were prepared in an identical manner except that the most coronal section was 0.5 mm rather than 1 mm in depth to capture the tissue that had formed in utero (*139*), and this section was frozen and then freeze-dried. All demineralized dentine sections were then heated to allow the collagen to go into solution, frozen at −35°C, and freeze-dried. All the resulting collagen samples were weighed into tin capsules. All samples were measured in duplicate by combustion in a Thermo Flash EA 1112 and introduction of separated N_2_ and CO_2_ to either a Finnigan Delta plus XL or a Delta V via a Conflo III interface at the University of Bradford Stable Isotope Laboratory.

The collagen samples were interspersed throughout the run with both internal standards and international standards. Calibrated against these standards, the analytical error at 1 SD was ±0.2‰ or better. The C:N ratios of all samples were within the range of 3.1 to 3.5 recommended by van Klinken (*140*) as a measure of collagen quality.

Each of the dentine δ^13^C and δ^15^N collagen profiles was plotted against the midpoint of the approximate age at which each section formed on the basis of (*86*) and as described in (*81*). All of these profiles were assessed by two of our research team members to identify any patterns that could denote nutritional distress. As detailed above, some profiles (Fig. 2) show opposing covariance of δ13C and δ15N that reflect distinct periods of nutritional stress after an individual was in a relatively stable state and before transitioning to a stable state. For some individuals, signs of nutritional stress at the beginning of a tooth profile appeared to be resolving, with δ15N decreasing from a high value as δ13C increased. For others, we see at the end of a profile a rising δ15N and flat or declining δ13C suggesting the onset of nutritional distress that had not yet peaked or resolved. Patterns in an early-forming part of a tooth that show both δ^15^N and δ^13^C values concurrently decreasing may reflect the effect of breastfeeding and weaning and can be distinguished from the nutritional stress pattern. The numbers of individuals with and without isotopic profiles indicating nutritional stress are shown in Table 1.

### Periosteal new bone formation

For this study, the first author scored the presence of PBNF on the tibia, as this bone is often well-preserved in archaeological contexts; we use data from the left tibia to maximize sample sizes for analyses. The first author assessed only the anterior surface of well-preserved tibiae for the presence of PNBF and, additionally for non-adult individuals, assessed only bone at least one centimeter from the epiphyseal growth plates to avoid mistaking normal growth processes for PNBF. The first author identified PNBF macroscopically under good lighting and scored it as present if there was a distinct patch of bone laid down on the surface of the diaphysis. We scored individuals with well-preserved anterior left tibiae that lacked PNBF as “absent” and those individuals missing left tibiae or with postmortem damage that precluded evaluation of the anterior tibiae as “unobservable” for PNBF. We exclude individuals scored as unobservable from analyses of PNBF. Table S3 in the Supplementary Materials displays the distribution of PNBF by time period and age group; of the 275 individuals included in our study, we have data on PNBF for *n* = 144 people.

### Statistical analyses

#### Survival and hazard analysis of the effects of nutritional stress

Survivorship and risks of mortality can be used as measures of underlying general health in living and past populations. For this study, we use Kaplan-Meier survival analysis and Cox proportional hazard analysis to assess how survivorship and mortality, respectively, differed between individuals with and without evidence of exposure to severe nutritional stress during childhood. For these analyses, we included all individuals (*n* = 268) for whom we could both estimate age and analyze dentine δ^13^C and δ^15^N collagen profiles. We compared survivorship between individuals with signatures of nutritional stress versus those without such signatures using data pooled across the entire study period (approximately 1000 to 1540 CE). We use point estimates of age and model nutritional stress as a covariate (those without signatures of nutritional stress were coded as “0,” and those with signatures of nutritional stress were coded as “1”). When we initially included data from people of all ages, Kaplan-Meier survival analysis and Cox proportional hazard analysis did not reveal any significant differences between people with and without signatures of severe childhood nutritional stress. However, we observed a crossing over of survival functions in the 30s. Specifically, the survival functions suggest that survivorship was higher for individuals with evidence of childhood nutritional stress up into the fourth decade of life, but then the trend reversed across older ages. Therefore, we performed further analyses separately for those individuals who died at ages younger than 30 years and those who died at 30 years of age and older. Individuals aged 30 years and older were included as right-censored data in the analysis of those younger than 30 years, as individuals 30+ years of age at death had survived past the age of 30, and thus provide some information about survival across age 0 up to 30 years and should be included in the analysis of differences in survivability at those younger ages. For the analysis of survivorship below the age of 30 years, individuals younger than 30 years were coded as having died between the ages of 0 and 29.99 years, and those aged 30 years and above were coded as not having died during this age range.

We used the same coding protocol for Cox proportional hazard analyses (no signature of nutritional stress = 0, signature of nutritional stress = 1) to evaluate how hazards of death differed between people with and without evidence of severe childhood nutritional stress. The semiparametric Cox model does not require the specification of the baseline hazard function and tests the null hypothesis that the covariate (in this case, nutritional stress) has no effect on the hazard, with the reported hazard ratio indicating the change in risk of death associated with a unit increase in the covariate. We performed analyses separately for individuals younger than 30 years and those older than 30 years across the entire study period.

We acknowledge that circa–Black Death and post–Black Death individuals are overrepresented in our study. Circa–Black Death people make up 28% of the overall number of individuals included in this study and about 35% of the individuals who are assigned to a specific time period within the general medieval period; post–Black Death individuals make up 29% of the overall total in this study and about 35% of those assigned to a specific time period (Table 1). To determine whether our findings are biased by unequal numbers of individuals across the time periods, and specifically biased toward patterns that occurred around the time of or after the Black Death, we examined survivorship and hazards of death associated with nutritional stress within each time period (early pre–Black Death, late pre–Black Death, circa–Black Death, and post–Black Death).

#### Analysis of patterns of PNBF

We compare differences in frequencies of PNBF between individuals with and without signatures of childhood nutritional stress using chi square tests.

#### Temporal trends in nutritional stress

We tested temporal differences in the prevalence of people with signatures of childhood nutritional stress, across all periods in our study (1000 to 1200, 1200 to 1250, 1250 to 1350, and 1350 to 1540 CE) using chi square tests.

We performed all analyses in SPSS v 29. We specified a priori an α value of <0.1. As in previous work [e.g., (*141*)], we chose this α level because we are working with relatively small sample sizes, and use of a lower α level might be unnecessarily conservative and lead us to ignore trends that are potentially meaningful.
