# Supplementary material for: Childhood nutritional stress and later-life health outcomes in medieval England: Evidence from incremental dentine analysis
Source: Sci Adv. 2025 Jul 30;11(31):eadw7076. doi: 10.1126/sciadv.adw7076 (PMC12309679; doi:10.1126/sciadv.adw7076)
Supplement: Supplementary file 1 — Table S3 Legends for tables S1 and S2 [file sciadv.adw7076_sm.pdf]

Science Advances
AAAS

Supplementary Materials for

Childhood nutritional stress and later-life health outcomes in medieval England: Evidence from incremental dentine analysis

Sharon N. DeWitte *et al.*

Corresponding author: Sharon N. DeWitte, sharon.dewitte@colorado.edu

Sci. Adv. **11**, eadw7076 (2025)
DOI: 10.1126/sciadv.adw7076

The PDF file includes:

Table S3
Legends for tables S1 and S2

Other Supplementary Material for this manuscript includes the following:

Tables S1 and S2

Table S3: Distribution of periosteal new bone formation (PNBF) by age group and time period. Note that for this table the "General Medieval (1000-1540 CE)" time period includes only those individuals who could not be assigned to any of the other specific time periods (Early pre-Black Death, Late pre-Black Death, Circa Black Death, or Post-Black Death).

Time Period	Age Group	PNBF Absent	PNBF Present
**General Medieval (1000-1540 CE)**	**<30**	10	4
	**30+**	2	12
**Early Pre-Black Death (1000-1200 CE)**	**<30**	1	2
	**30+**	1	2
**Late Pre-Black Death (1200-1250 CE)**	**<30**	11	10
	**30+**	3	4
**Circa Black Death (1250-1350 CE)**	**<30**	20	7
	**30+**	8	2
**Post-Black Death (1350-1540 CE)**	**<30**	21	8
	**30+**	5	9
	**unknown**	1	1

Table S1: Raw incremental dentine isotope data. These are the data used to produce dentine isotope profiles to identify patterns of opposing covariance indicative of nutritional stress. Data are organized by individual, and include isotope values for each section of each tooth sampled from each individua, information about which tooth was sampled, the growth period for that tooth, and the approximate age for each tooth section analyzed.

Table S2: Data used in analyses. These data include site name, context number for each individual, time period, burial type, estimated age, periosteal new bone formation (PNBF scores), and nutritional stress scores. Time period: 0 = 1000-1200, 1 =1200-1250, 2 = 1250-1350, 3 = 1350-1540. PNBF Left Tibia: = unobservable, 1 = absent, 2= present. Nutritional stress: 0 = no, 1 = yes.
